# Supplementary material for: Soil properties and root traits jointly shape fine-scale spatial patterns of bacterial community and metabolic functions within a Korean pine forest
Source: PeerJ. 2021 Feb 26;9:e10902. doi: 10.7717/peerj.10902 (PMC7919533; doi:10.7717/peerj.10902)
Supplement: Supplemental Information 1 [file peerj-09-10902-s001.docx]

Table S1 Summary statistics for soil bacterial community composition and metabolic functions, root traits and soil parameters (n=53).

| Variables | Mean | SE | Min | Max | CV/% |
| --- | --- | --- | --- | --- | --- |
| Microbial community properties |  |  |  |  |  |
| OTUs | 2901 | 32 | 2311 | 3402 | 8.02 |
| Bacterial diversity (H') | 6.28 | 0.029 | 5.73 | 6.84 | 3.39 |
| Metabolic activity (AWCD 72h) | 1.00 | 0.03 | 0.51 | 1.57 | 28.8 |
| Metabolic diversity(H') | 2.59 | 0.036 | 2.19 | 3.35 | 11.7 |
| Root traits |  |  |  |  |  |
| Biomass (kg m^-2^) | 0.268 | 0.016 | 0.042 | 0.513 | 44.2 |
| Length (km m^-2^) | 5.67 | 0.518 | 0.042 | 17.7 | 66.5 |
| SRL (m g^-1^) | 46.3 | 2.03 | 21.0 | 102.5 | 32.0 |
| RTD (g cm^-3^) | 1.19 | 0.05 | 0.55 | 2.96 | 30.5 |
| Diameter (mm) | 0.45 | 0.02 | 0.09 | 0.82 | 26.9 |
| Soil properties |  |  |  |  |  |
| pH | 5.54 | 0.04 | 5.05 | 6.59 | 5.9 |
| TN (g kg^-1^) | 8.95 | 0.36 | 4.27 | 14.7 | 29.2 |
| SOC (g kg^-1^) | 111.4 | 4.97 | 54.1 | 196.4 | 32.5 |
| C/N | 12.4 | 0.015 | 10.2 | 16.2 | 11.1 |
| DOC (mg kg^-1^) | 212.2 | 8.98 | 131.0 | 395.0 | 30.8 |
| DON (mg kg^-1^) | 25.2 | 2.30 | 5.34 | 88.4 | 66.3 |
| POC (g kg^-1^) | 5.09 | 0.36 | 1.70 | 15.3 | 52.0 |
| PON (g kg^-1^) | 0.20 | 0.01 | 0.06 | 0.60 | 50.9 |

CV, coefficient of variation; SE, Standard error of mean; H', Shannon index; SOC, soil organic carbon; TN, total nitrogen; C/N, SOC/TN; DOC, dissolved organic carbon; DON, dissolved organic nitrogen; POC, particulate organic carbon; PON, particulate organic nitrogen; SRL, specific root length.

Table S2 Pearson’s correlations between environmental variables

|  | pH | SOC | TN | CN | DOC | DON | POC |
| --- | --- | --- | --- | --- | --- | --- | --- |
| SOC | .173 |  |  |  |  |  |  |
| TN | -.050 | .937^**^ |  |  |  |  |  |
| CN | .593^**^ | .424^**^ | .093 |  |  |  |  |
| DOC | .038 | .757^**^ | .701^**^ | .358^**^ |  |  |  |
| DON | -.038 | .327^*^ | .279^*^ | .222 | .474^**^ |  |  |
| POC | .144 | .270 | .194 | .227 | .358^**^ | .397^**^ |  |
| PON | .106 | .156 | .134 | .078 | .304^*^ | .359^**^ | .897^**^ |

^*^, *p* < 0.05; ^**^, *p* < 0.01.

Table S3 Model summary for the stepwise multiple regression of metabolic function on bacterial groups and environmental variables

|  | Adj.r^2^ | | Contribution of the individual environmental predictor (%) | | | Contribution of the bacterial groups | | |
| --- | --- | --- | --- | --- | --- | --- | --- | --- |
|  | Full model | | pH | DOC | Others | | Correlated bacterial groups | Total contributions (%) |
| Average | 0.792 | | 48.1 | 24.9 | 2.27  (SRL) | | Proteobacteria_unclassified, Verrucomicrobia_Incertae_Sedis,  JG30-KF-CM66, Thermomicrobia, Planctomycetacia | 24.8 |
| Carbohydrates | 0.736 | | 52.6 | 17.1 |  | | Proteobacteria_unclassified, S-BQ2-57_soil_group, Planctomycetacia, Verrucomicrobia_Incertae_Sedis, Thermoleophilia, JG30-KF-CM66 | 30.3 |
| Carboxylic acids | | 0.736 | 39.3 | 32.1 |  | | Bacteroidetes_unclassified, Gemmatimonadetes,  JG30-KF-CM66, Anaerolineae, Planctomycetes_unclassified | 28.7 |
| Amino acids | 0.572 | | 37.8 | 24.8 | 6.8  (SOC) | | Bacteroidetes_unclassified, JG30-KF-CM66, Bacteria_unclassified。 | 30.6 |
| Polymers | 0.897 | | 53.0 | 23.3 | 1.34 (Biomass) | | Deltaproteobacteria, Pla3_lineage, Chloroflexi_unclassified, JG30-KF-CM66, KD4-96, Armatimonadetes_unclassified, Parcubacteria_unclassified, Anaerolineae, vadinHA49 | 22.4 |
| Miscellaneous | 0.714 | | 28.9 | 17.7 |  | | Proteobacteria_unclassified, Verrucomicrobia_Incertae_Sedis, Thermoleophilia, Actinobacteria_unclassified, Planctomycetacia, TA06_unclassified | 53.5 |
| Amines | 0.597 | | 57.8 | 25.5 |  | | SPOTSOCT00m83 | 16.8 |


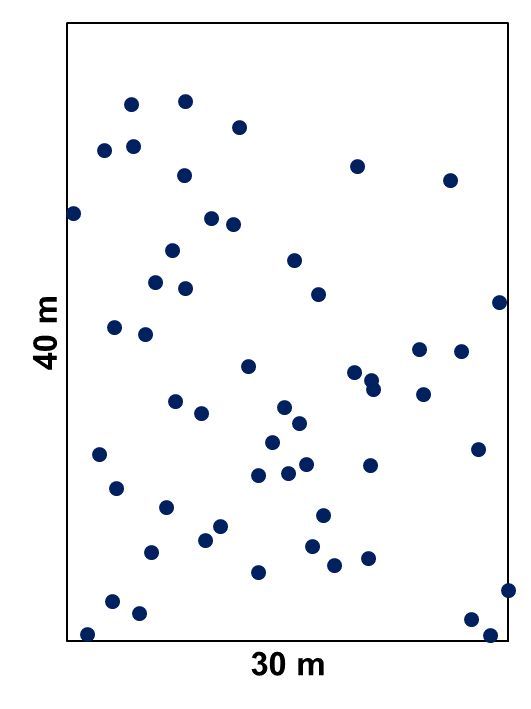


Fig. S1 The soil sampling scheme.
